# A Cross-sectional Analysis of Facebook Comments to Study Public Perception of the Mass Drug Administration Program in the Philippines

**DOI:** 10.34172/ijhpm.2020.55

**Published:** 2020-04-22

**Authors:** Ryan V. Labana, Veeranoot Nissapatorn, Kristel Joy S. Cada, Khristine L. Sandoval

**Affiliations:** ^1^Department of Biology, College of Science, Polytechnic University of the Philippines, Sta. Mesa, Philippines.; ^2^School of Allied Health Sciences (Southeast Asia Water Team), Walailak University, Nakhon Si Thammarat, Thailand.; ^3^Research Excellence Center for Innovation and Health Products (RECIHP), Walailak University, Nakhon Si Thammarat, Thailand.; ^4^Biology Department, College of Arts and Sciences, New Era University, Quezon City, Philipppines.; ^5^College of Arts and Sciences, National University-Laguna, Calamba Laguna, Philippines.

**Keywords:** Albendazole, Mass Drug Administration, Public Health Practice, Philippines, Social Media

## Abstract

Mass Drug Administration (MDA) of albendazole is being implemented in the Philippines to eliminate soil-transmitted helminthiasis (STH) among school-age children (SAC). The first round of MDA for the school year 2019-2020 was suspended in the province of Surigao del Norte due to a reported death of a student after deworming. It was broadcasted on a national television and the story was then posted on Facebook. We used structured and simple nature of social media research to study public perception of the MDA program after the controversial issue in the Philippines. The news story was assessed, and the Facebook comments were analyzed. A large portion of the Facebook comments expressed a declining trust of the Filipinos toward MDA. The negative impact could be attributed to the public-initiated discussion and sharing of comments with no solid evidence in Facebook. This study showed a possible threat to the successful implementation of the program if not properly managed. The Department of Health (DOH) should be able to cope with the developing landscape of public perception during the era of social media.

## Background

Soil-transmitted helminthiasis (STH) remains a major public health issue in the Philippines. It can cause anemia, malnutrition, impaired physical and cognitive development in children, and sometimes early death.^1-3^ Recent data showed STH prevalence of >20% among school-age children (SAC) in 13 out of 17 regions in the country, which are mainly caused by *Ascaris lumbricoides, Trichuris trichiura,* and hookworms.^1^ Through the recommendation of World Health Organization (WHO) to deworm SAC in areas with ≥20% prevalence of intestinal worms,^3^ the Department of Health (DOH), the Department of Education (DepEd), and their partners are conducting the Mass Drug Administration (MDA) every January and July. The DepEd Order No. 10, s. 2016 targets 85% of all SAC to get dewormed during these months. The goals of the program include (1) reduce students’ absenteeism caused by worm infection, (2) maximize the learners’ physical and mental potential, (3) free the learners from illnesses and nutrient deficiencies, and (4) reduce the transmission of the disease in the community.^4^

For the school year 2019-2020, the first round of MDA commenced on July 8 and was due to run until July 26, 2019 but health authorities suspended its completion in the province of Surigao del Norte due to a reported death incident of a student after deworming. A news program *Bandila* (Flag) of ABS-CBN, a major TV network in the Philippines, broadcasted and posted the story on its Facebook account on July 31, 2019. It received an influx of comments and reactions from the netizens (the people who use the internet) until they removed it from their page on the following day. ‘Showbiz Philippines,’ an independent and unaffiliated Facebook page with 6800+ followers, was able to pick-up the video and repost it in their Facebook page where netizens continuously expressed various comments and reactions.^5^

### The Television News Story Posted in Facebook

The title of the Facebook post which is in a video format is *“Estudyantepatay, 7 iba pa naospitalmataposumanongpainumin ng pampurga”*(A student died, 7 others hospitalized after deworming). According to the ABS-CBN report, there were more than 300 students of San Isidro Elementary School in Surigao del Norte, Philippines who were given albendazole during the MDA on July 23, 2019. Based on the report, one of the students experienced loss of vision and stomachache, 5 hours after taking the medicine. The student was immediately brought to the hospital but succumbed to death prior to the admission. According to the student’s school adviser, the student informed her of experiencing stomachache last June; however, the student was already feeling fine on the day of MDA. Also, the students took their meals before the drug administration as a guideline practiced in the school. After briefing, their parents agreed to the protocol and signed on behalf of their children in the consent forms. Of this, the DOH investigated the cause of death and the detailed information during hospitalization of the students. DOH noted that the medicine will expire on April 2021, however, albendazole samples were still sent for the toxicity test under the Philippines Food and Drug Administration (FDA). Meanwhile, the victim’s parent awaited to be informed of the autopsy results. Other parents were aware of the incident and some of them took serious precautions of their children to participate again in the MDA.^5^

### Social Media as a Catalyst for Evolving Public Health Insights

There is a growing number of social media research in the field of public health.^6^ It is a valuable and innovative practice because it augments public health capabilities in terms of monitoring adverse health events and health promotion, among others.^7^ In a viewpoint published in the *Journal of Medical Internet Research*,^8^ understanding the impact of health misinformation or disinformation in shaping public’s attitudes, beliefs, and behavior toward health promotion and practices must be highly considered in social media research nowadays. The characteristics of both the sources (social media platforms) and the recipients (public) of the information that affect message receptivity should also be recognized. Health ‘misinformation’ means sharing of false information without an intention to cause harm while ‘disinformation’ means creation and advertently sharing wrong information to mislead people and cause ambiguity.^9^ These are abundant in Facebook and they become a major platform in sharing opinion and compelling (yet possibly distorted) stories about people’s experiences. The easy access to reporting and blogging of various groups using this platform limits the quality of information it provides to the public. These forms of communication can bring potential harmful effects to the people’s health and well-being. Public health administrators should distinguish the type of health literacy education program that can curve the public consumption of health misinformation and/or disinformation.^10^

In the Philippines, the DOH, which is the chief implementer of MDA as a major STH control strategy, was challenged for several times in preventing the eroding public trust of the Filipinos in their various public health programs. The department has remarkably improved their basic public health services for the Filipinos, but the rise of social media usage challenges them to anticipate in this era of change.^11,12^ In 2018 for instance, Dengvaxia-scare, which was further intensified by social media, affected the implementation of the national mass deworming among SAC.^12-14^ Dengvaxia is the first dengue vaccine developed by the Sanofi Pasteur, one of the biggest multinational pharmaceutical companies. Dengvaxia was used in the vaccination campaigns in the Philippines among SAC, but soon encountered controversies due to Dengvaxia that may cause severe form of dengue if inoculated to children with no history of the disease. The Philippine FDA momentarily suspended its distribution in the country and the ‘viral rumors’ left most Filipino parents in doubt of the safety of vaccination.^12^ Based on the recent scenario, DOH confirmed that at least 60% of the parents opposed the deworming program.^13^ Furthermore, in a study conducted in Cagayan Valley, teachers estimated ≤50% decline of the parents giving consent to deworm their children because of the safety issue.^14^ DOH announced that Dengvaxia-scare was already over in July 2019 based on the result of a national survey, but they were not able to specify if it was a result of their information drive via mainstream media, to educate the public on the importance of vaccines.^15^ The recent news on the death of a student and hospitalization of seven others after deworming has gained much attention from DOH in tackling related public health problems. The goal of this paper is to analyze the quality of information brought by the news story posted in Facebook, determine its impact on the public perception of MDA, and obtain possible lessons learnt.

## Methods

### Social Media Research Approach

We used structured and simple analysis of social media research that aimed to answer defined questions.^16^ We arrived at 3 research questions (RQs) in this study: (RQ1) what are the attributes of the news story posted in Facebook? (RQ2) what is the impact of the news story on the public perception of MDA? (RQ3) what are the implications of the quality of news story to its impact on the public perception?

### News Story Assessment

We used the guide of evaluating news stories developed by Zimdars, which seeks for its reliability by assessing several criteria in terms of (1) type of article, (2) domain, (3) evidence, (4) publishers and journalists, (5) sources, and (6) aesthetics. The assessment was guided by several indicators per criteria as either ‘in favor of credibility’ or ‘not in favor of credibility.’ The tool was licensed under a Creative Commons 4.0 International License and University of Texas Library.^17^

### Gathering and Analysis of Facebook Comments

We utilized the data gathering procedure on cross-sectional analysis of Facebook comments to study public perception by Tan et al^18^ with modification. We retrospectively reviewed all comments of the netizens in response to the news story. We extracted all the comments to the posting from July 31 to August 12, 2019 using a ‘Facebook graph explorer,’ an application programming interface that retrieves data from Facebook and transports it to MS Excel for analysis. Extracting of comments was completed on August 12, 2019 as the hype of the news has already declined with the new updated news surfacing on the Facebook page. Most Facebook comments were in Filipino and few comments were in a Visayan language. These were translated to English using ‘Google Translate’ and were manually cross-checked by the researchers for accuracy of translation when deemed needed. All the researchers can understand and speak English language, three of them can understand Filipino and Visayan languages, and one of them is a native of Visayas. To ensure the reliability in interpretation of themes, two researchers conducted the coding process, the other two researchers reviewed the codes for accuracy, and all participated in the discussion and further analysis. The comments were based on the unbiased public reaction on social media without involvement of the family of the patients and without the use of biological samples. Therefore, the ethical approval was not required for this study.^18^

We employed a passive analysis of data, which involved the study of information patterns as reflected on the Facebook comments.^18,19^ We first extracted Facebook comments and group them into pre-set categories using MAXQDA Analytics Pro (VERBI GmbH; Berlin, Germany). The rational themes that emerged include (1) positive comments, (2) negative comments, (3) questions, (4) unsolicited suggestions, (5) tagged comments, and (6) unknown. The comments of each Facebook user were analyzed in a non-mutually exclusive manner. In some cases, comments were categorized as positive, negative, and unsolicited suggestions should they contain information which crosses these domains. Similarly, comments that contained multiple points in varying categories were each considered individually. We used our second research question (RQ2) as a guide in coding. ‘Positive comments’ reflected with a sustained trust of netizens in MDA, ‘Negative comments’ reflected as disagreement to the conduct of MDA, ‘Questions’ reflected as a sort of inquiry about the issue, and ‘Unsolicited suggestions’ are recommendations pertaining to the program implementation. ‘Tagged comments’ refer to the feature of Facebook that attaches the name of other Facebook users, creating a link to their Facebook profile in the comment section. It calls the attention of the tagged Facebook user(s), which allows the spread of information. This mechanism is essential in making the issue ‘viral’ and could possibly solicit further public perception; however, the responses of the tagged Facebook users are not implicit, so they were not included in the analysis. ‘Unknown comments’ on the other hand included emoticon (a graphical expression embedded in the comment section as an *emoji*) and/or a picture file in GIF format used to provide emotional information. We also excluded this type of comments in the analysis to delimit our findings on the grammatical elements and valence of the sentiments reflected in the Facebook comments. Axial coding was done in comments that cross the four categories, a procedure for interconnecting the categories. Lastly, we conducted selective coding, where we built a story that connects the categories for a discursive set of theoretical propositions.

In order to further analyze the data, we have used the well-known Health Belief Model (HBM) as a guide. HBM is a model used by the policy makers in developing health education messages and campaigns.^20^ This model is now being used by various researchers to analyze social media posts about public health issue.^21,22^ We modified HBM in our study wherein we identified the number of negative comments to determine the ‘*perceived threats*’ of participating in MDA, the number of positive comments to determine the *‘perceived benefits’* of participating in MDA, and the number of questions and unsolicited suggestions as the *‘perceived barriers’* in the successful implementation of MDA. We used these constructs to determine the prevailing impact of the news story posted in Facebook to the public perception towards MDA.

## Results

### RQ1: What Are the Attributes of the News Story Posted in Facebook?

The article was a news story in a video format that answered the 5 W’s and H questions. When promoted in social media, the title of the news story accurately reflects its content. The journalist, the media outfit, and the Facebook page where it was first posted, belongs to a large media conglomerate in the Philippines that has earned credibility for years. In terms of ‘evidence,’ the news story produced verified and actual video, audio, and photographs of the persons involved and eyewitnesses. The weakness of the news story, as perceived by the researchers, was the lack of transparency on the probability of various consequences, the risk of taking the medicines, and the clear yet simple demonstration of mechanisms of albendazole in the body. These pieces of information would be more helpful if provided upfront because social media is consumed by the public in a very fast pace. It could also be a part of the regular updates as the story unfolds through presentation of the result of the autopsy (postmortem), which could be incorporated in the follow-up news story. Unfortunately, there was no follow-up news story was found after the original news story was subsequently deleted by ABS-CBN.

### RQ2: What Is the Impact of the News Story on the Public Perception of MDA?

As of August 12, 2019, the video has garnered 879 000 views and 1200 comments. Of the 1200 comments, 194 (16.2%) were categorized as ‘Negative comments,’ 115 (9.6%) as ‘Unsolicited suggestions,’ 33 (2.8%) as ‘Positive comments,’ and 13 (1.1%) as ‘Questions.’ There were 61 (5.1%) comments categorized as ‘Unknown’ and 784 (65.3%) as ‘Tagged comments’ (Table 1).

**Table 1:** Number of Comments Per Identified Theme

**Themes**	***N***	**%**
Positive comments	33	2.8
Negative comments	194	16.2
Questions	13	1.1
Unsolicited suggestions	115	9.6
Unknown	61	5.1
*Tagged comments*	784	65.3
Total	1200	100

Of the 1200 comments, only 33 (2.8%) expressed their confidence on the drug safety used in MDA program after the reported death incident of a student associated to deworming. Most of these comments provided generally favorable testimonies regarding the safety of albendazole as also experienced in their areas. Some comments included:

*“Here in our area, 100% of the students took the medicine in front of the teacher, and no one got harmed.”*

*“There are 98% of SAC in our area who took the medicine, and they are all fine.”*

*“In our place, even the nurse and parents took the medicine. Nothing bad happened to all of us.”*

Few comments expressed an idea that the death incident of the student after deworming was possibly an isolated case. Example quote:

*“Maybe, the child has other medical condition that’s why she died. So far, we don’t have bad incident in our area.”*

There was one comment that expressed firm standpoint about the safety of albendazole as opposed to the claims of other commenters that albendazole causes erratic movement of the worms after they were knocked-out, which can subsequently cause their migration to other body parts and further cause complications.

*“With the new drug, worms will not come out erratically. Make a research to confirm my statement, for your judgement.”*

One commenter was found consistently responding to the negative concerns of others. She was trying to educate people about the safety of albendazole and was persistent that the death incident could be an isolated case.

The 194 (16.2%) negative comments out of 1200 were further sub-categorized according to themes that arose (Table 2). Of these, 92 (47.4%) expressed their lack of confidence of the drug safety for various reasons. Some of the commenter even expressed anger toward the reported incident. They also perceived free medication from the responsible agency (RA) with low quality and near expiration, if not already expired. Some comments include:

**Table 2:** Breakdown of “Negative Comments” to the News Story

**Negative Comments**	**N**	**%**
I do not trust in the health programs of RA	92	47.4
I start doubting deworming in school	56	28.9
I do not trust in the health program of RA in the Philippines after the Dengvaxia issue	24	12.4
I do not trust deworming in school because of the adverse effects that cannot be managed by the teachers	12	6.2
We also experienced negative impact of deworming in our area	10	5.2
Total	194	100

Abbreviation: RA, responsible agency.

*“Knowing that it is coming from RA, it must be a cheap type of drug.”*

*“If it is coming from RA, expect that it is already expired.”*

*“That is why I do not allow my child to take free medication because these are medicines that are about to expire.”*

Some of the comments expressed no confidence on the integrity of the RA to administer MDA among SAC.

*“Teachers should not be entrusted with deworming of children because they are not the authorities of health.”*

*“For the fact that they (teachers) ask first the parent to sign a waiver, there might something wrong with it.”*

There were 56/194 (28.9%) comments that expressed their lack of confidence about the possible outcome of deworming in school due to the drug safety used in the MDA program among SAC. Some of these commenters have already signed the parent’s consent for MDA in July and they showed serious concerns on the outcome of their decision. They further expressed their acceptance with more cautions for their children to participate in the future program. Others are comforted knowing that their children have not yet taken the medication despite their approval. They would also be more concerned about participating health interventions provided with the proper guideline information of their SAC. Some comments included:

*“Thank you for the information. I will not allow my child to take the tablet from now on.”*

*“I’ll back out with my signed consent.”*

*“I still did not administer the medicine from the center. I am now afraid to do it.”*

Other negative comments (*N*= 24/194; 12.4%) were found associating with the vaccination program (Dengvaxia) which happened in 2018. They expressed their hesitance of participating in MDA due to the impact of Dengvaxia-scare even though it is not directly related to MDA program of DOH. There were also claims of unreported negative incidence, even death of children, after deworming in other regions.

*“There are also other students who experienced it here in our area in Mandaluyong City (Metro Manila). Until now, the child is in the hospital with severe stomachache.”*

*“Here in our place in Negros, someone also died after deworming but was not reported to the authority.”*

*“Like what happened here in Quezon province, the tablet knocked the worms out, so they behaved erratically until they caused holes in the intestine.”*

Interestingly, it was also evident in the comments that there are varying levels of knowledge, attitudes, and practices of the Filipinos toward deworming their children. There were 115 (9.6%) unsolicited suggestions on perceived effective management of intestinal worms of the children. These suggestions were presented in Table 3.

**Table 3:** Breakdown of “Unsolicited Suggestions” Related to the News Story

**Unsolicited Suggestions**	**N**	**%**
Should have a proper consultation with the physician before the administration of the drug	54	47.0
Deworming should be done in proper health facility (not in school)	21	18.3
Should use other natural remedies for deworming	18	15.7
Should ensure the child has eaten first before taking the medicine	10	8.7
The nurse should take the medication first to see if there are any side effects (the researchers considered the comments having a tone of sarcasm)	5	4.3
The child should take the table with empty stomach	3	2.6
"Please share this post so everybody is aware"	2	1.7
Deworming should not be done in rainy season because worms are awake during this time	1	0.9
The drug should be tried first in animals if it is effective before administering to humans	1	0.9
Total	115	100

There were 54/115 (47.0%) suggestions on the importance of proper consultation with the doctor before the administration of albendazole.

*“A child should take something that paralyze the worm first before taking the medicine. Also, it should not be given to a child with flue and cough. That is what the doctor said.”*

*“The dosage of the medicine should be suitable to the weight of the child. Ask your doctor.”*

There were also comments (*N*= 21; 18.3%) that empathized teachers for being involved in the issue. They expressed that corresponding duties should be taken by the RA in the country. With this, they suggest not to conduct the deworming in school anymore. Some comments include:

*“It is an additional task for us, teachers. It should not be entrusted to us anymore.”*

*“The drug should be given to the parent and let the parent administer it.”*

On the other aspect, we have observed comments (*N*= 18/115; 15.7%) that suggest natural remedies for deworming as a safe practice. Of the 18 suggestions, 13 mentioned about* ‘Ipil-ipil’ (Leucaena leucocephala)* as an effective deworming agent. Some of the comments include:

*“When we were children, we take the fruit of Ipil-ipil to deworm and it is effective.”*

*“Better take fruits and boiled ‘Tanglad’ (Cymbopogon citratus) every day until the fever subsided.”*

*“Ripe jackfruit (Artocarpus heterophyllus) is effective for deworming… It is natural so it is safe.”*

Others (*N*= 13/1200; 1.1%) asked unprecedentedly questions in the comment section. Some of these questions were answered anonymously but most were left unattended. The questions included:

- *“Why did it happen?”*
- *“Was it caused by an overdose?”*
- *“Will natural remedies kill the worm, or will they let the worm out alive?”*

We have plotted the Facebook comments in a Mind Map (Figure) to present the prevailing ideas about the public perception of the MDA in the Philippines that have surfaced after watching the news story and we utilized HBM principle to elucidate meanings. The size of the circles in our model determines the frequency of comments that entails a theme. It can be observed that the frequency of negative comments was greater than the frequency of positive comments, which implies a higher ‘*perceived threats*’ of participating in MDA than the *‘perceived benefits’*despite of the statement of the DOH undersecretary that the incident was an isolated case.^23^ We have also identified few questions and unsolicited suggestions, which can be related to lack of right information about MDA. We consider them as a *‘perceived barrier’* in the successful implementation of the program. Overall, we theorize that the Facebook post has a negative impact to the public perception toward MDA program and should therefore be targeted by the policy makers in the public health promotion.

**Figure:**
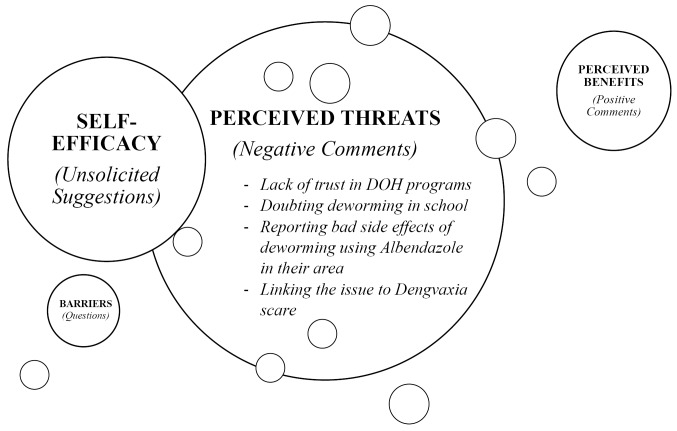
Mind Map Presentation of Themes on Public Perception of the MDA of Albendazole in the Philippines After Watching the News Story. Abbreviations: DOH, Department of Health; MDA, Mass Drug Administration.

### RQ3: What Are the Implications of the Quality of the News Story to its Impact on the Public Perception?

The news story is perceived by the researchers as ‘newsworthy’ based on the criteria used in this study including type of article, domain, evidence, publishers/journalists, sources, and aesthetics. Despite some weaknesses that we have identified, the news story generally met the news values commonly considered in the journalistic standpoint.^24^ However, the impact of the news story was found in this study as negative when posted in Facebook. The negative impact could be attributed to the public-initiated discussion and sharing of comments with no solid evidence in the social media platform, which enable the formation of a collective public opinion without any restrictions. Several misinformation and disinformation about MDA may not be observed in the news story but they appear in the Facebook comments. These health misinformation and disinformation were consumed by the public as conceivably accurate and they cause further damaging effect to the public perception toward MDA. The news values were even more crucial because it concerns the SAC. Although it requires further investigation, but the impact could have been lesser if it concerns gambling, addiction, and other public health issues involving a minority group of people.

## Discussion

This qualitative analysis provides a glimpse to the current perception of the Filipinos toward MDA (and other government programs in general) as reflected on the Facebook comments to the news story about the death of a student after deworming. Its scope is limited because the source of data is not a major Facebook page run in the Philippines contrasting ABS-CBN News that usually garners a million of views and thousands of comments. The removal of the news story in the major Facebook page might be a right move to eliminate ‘viral rumors’ that can cause massive scare while DOH are still probing the incident. Despite the lesser impact of this controversial issue as compared to Dengvaxia-scare in 2018, our analysis still shows an interesting idea of how Facebook comments reflect and affect the perceptions of the Filipinos toward a certain public health issue.

Research on the influence of social media to the delivery of public health program is limited in the Philippines but it is an emerging topic across the globe. One concern of the public health administrators nowadays is the spread of health-related misinformation and disinformation on social media. In 2015, the DOH in the Philippines investigated reported adverse events following mass drug administration (AEFMDA) using albendazole through an event-based surveillance system in Zamboanga Peninsula on the island of Mindanao, Philippines. From 12 million children subjected to national MDA, 77 cases of AEFMDA were identified by the DOH with abdominal pain, headache, and vomiting. There were no deaths reported but 39 cases were hospitalized. After thorough investigation, the DOH considered the case as a result of epidemic hysteria – a group of symptoms for organic illness with no cause(s). It was triggered by the spread of a text message in the region regarding a false report of deaths among children who took an expired deworming tablet distributed by DOH. The media coverage aggravated the incident so the DOH/DepEd used risk communication to pacify the public.^6^ In our study, many the commenter lost their confidence in MDA without validating other possible reasons of the incident. The uncontrolled broadcasting of negative news about MDA in Facebook might continuously exacerbate the delivery of the health package program as Filipinos tend to indiscriminately consume the news story they watch in Facebook.

If the news about MDA in the Philippines is not carefully managed, it is possible to continuously lose the confidence of the Filipinos toward this major intervention of eliminating STH in the country. DOH should be coordinating intently with the major news programs when a public health issue arises in order to properly manage the broadcasting that is easily consumed and shared by the people. DOH should also work together with the social media ‘influencer’ to educate them about various public health programs so they would create responsible contents in their social media pages. While the DOH is doing its best to control the damage of social media coverage, it would also be helpful if they spearhead trainings on responsible communications of health-related case reports. Also, it should conduct more studies about the good impact of deworming and share compelling stories on how it improved the over-all health in certain groups of people.

## Conclusion

The newsworthiness of a news story produced by the mainstream media may be distorted once it reached the social media platforms like Facebook. The unregulated exchange of comments and reactions toward the news story may become an avenue of sharing misinformation and disinformation regarding an important societal issue. This could bring harmful effects in the public health sector of a society. Health communication in the country should be revolutionized in a way it can reach the public easier, therefore, social media could be a promising platform in the current scenario. DOH should maximize the use of Facebook, Twitter, Instagram, and other social media platforms in the health care system including patient education, public health programs, and professional education because it is widely consumed by the Filipinos from all facets of life. This platform should serve the government for a common good and should not deteriorate existing programs that are helpful to the people. DOH should be able to cope with the evolving landscape of public perception during the era of social media.

## Acknowledgements

This research did not receive any funding but we thank the Commission on Higher Education for allowing us utilize some resources of our other research project: K to 12 Transition Program Management Unit, under the Discovery Applied Research and Extension for Trans/Interdisciplinary Opportunities (DARE TO) Grant-in-Aid 2017 (Project Number: DARETO2-043).

## Ethical issues

The primary data are Facebook comments from anonymous people and were based on the unbiased public reaction on social media without involvement of the family of the patients. With this, ethical approval was not required for this study.

## Competing interests

Authors declare that they have no competing interests.

## Authors’ contributions

RVL conceptualized the study and led in data gathering, analysis, and in writing the paper; RVL, VN, KJSC, and KLS worked on data gathering, analysis, and in writing the paper.

## Authors’ affiliations

^1^Department of Biology, College of Science, Polytechnic University of the Philippines, Sta. Mesa, Philippines. ^2^School of Allied Health Sciences (Southeast Asia Water Team), Walailak University, Nakhon Si Thammarat, Thailand. ^3^Research Excellence Center for Innovation and Health Products (RECIHP), Walailak University, Nakhon Si Thammarat, Thailand. ^4^Biology Department, College of Arts and Sciences, New Era University, Quezon City, Philipppines. ^5^College of Arts and Sciences, National University-Laguna, Calamba Laguna, Philippines.
